# Assessment of Renal Transplant Perfusion by Contrast-Enhanced Ultrasound after Switch from Calcineurin Inhibitor to Belatacept: A Pilot Study

**DOI:** 10.3390/jcm11154354

**Published:** 2022-07-27

**Authors:** Bilgin Osmanodja, Frédéric Muench, Alexander Holderied, Klemens Budde, Thomas Fischer, Markus Herbert Lerchbaumer

**Affiliations:** 1 Department of Nephrology and Medical Intensive Care, Charité–Universitätsmedizin Berlin, Charitéplatz 1, 10117 Berlin, Germany; frederic.muench@charite.de (F.M.); alexander.holderied@charite.de (A.H.); klemens.budde@charite.de (K.B.); 2 Department of Radiology, Charité–Universitätsmedizin Berlin, Charitéplatz 1, 10117 Berlin, Germany; thom.fischer@charite.de (T.F.); markus.lerchbaumer@charite.de (M.H.L.)

**Keywords:** kidney transplantation, calcineurin inhibitors, ultrasonography

## Abstract

Calcineurin inhibitors (CNIs) have improved short-term kidney allograft survival but are nephrotoxic and vasoconstrictive. Vasoconstriction is potentially reversible after switching from CNIs to belatacept. The kidney allograft shows optimal requirements for dynamic perfusion imaging using contrast-enhanced ultrasound (CEUS). We performed standardized CEUS in patients after switching from CNIs to belatacept for clinical indication to study the suitability of CEUS, in order to assess the effects of CNI cessation on kidney allograft perfusion. Eleven kidney transplant patients were enrolled from February 2020 until November 2020. Demographic, clinical, and laboratory parameters, as well as perfusion imaging, were assessed at baseline and 6 months after switching immunosuppression. Quantification of perfusion imaging on CEUS was performed using a post-processing software tool on uncompressed DICOM cine loops. After CNI cessation, estimated glomerular filtration rate increased by 4.8 mL/min/1.73 m^2^ (16%). Despite good quality of fit and comparable regions of interest in baseline and follow-up CEUS examinations, quantification of perfusion imaging showed a slightly improved cortical perfusion without reaching statistical significance after CNI cessation. This is the first study that systematically investigates the suitability of CEUS to detect changes of microvascular perfusion in kidney transplant recipients in vivo. No significant differences could be detected in perfusion measurements before and after CNI cessation.

## 1. Introduction

Calcineurin inhibitors (CNIs) have substantially improved short-term graft survival in kidney transplantation. However, CNIs have unfavorable effects on long-term outcomes of kidney transplant recipients (KTRs) due to potential nephrotoxicity and cardiovascular adverse effects [1,2,3,4,5].

CNI nephrotoxicity is a result of both reversible changes and irreversible damage to all compartments of the kidneys, including glomeruli, arterioles, and tubulo-interstitium. Acute CNI toxicity is caused by a variety of pathophysiological changes and consequentially leads to several histopathological findings: vasoconstriction of the afferent arterioles displayed by reduced vessel diameter, toxic effects on tubuli resulting in isometric vacuolization of the tubular cytoplasm, and thrombotic microangiopathy [2].

While acute CNI toxicity can cause dramatic impairment of kidney function, especially in the early post-transplant period, this has little impact on the long-term outcome due to its reversibility. On the contrary, chronic CNI toxicity plays an important role in chronic allograft injury and graft failure [6]. The complex mechanisms of injury have been studied mostly for cyclosporine but are regarded to be comparable for tacrolimus. In summary, a combination of CNI-induced hemodynamic changes, and direct toxic effects of CNIs on tubular epithelial cells, is thought to play a major role in chronic CNI toxicity. Histologically, arteriolar hyalinosis (nodular hyaline deposits in the media of afferent arterioles), interstitial fibrosis, and tubular atrophy, as well as glomerular sclerosis, are common features of chronic CNI toxicity.

Against the background of those shortcomings of CNIs, alternatives have been widely studied to improve long-term outcomes of KTR. Most recently, belatacept, a fusion protein linking the human IgG1 Fc fragment to the modified extracellular domain of cytotoxic T-lymphocyte-associated antigen 4, has shown promising results [7]. Belatacept selectively inhibits T-cell activation by blocking the CD28–CD80/86 costimulatory pathway. Patients receiving maintenance immunosuppression with belatacept have higher eGFR and less de novo anti-human leukocyte antigen (HLA) antibodies, but are more likely to develop acute T-cell-mediated rejection and rarely develop post-transplant lymphoproliferative disorders. Even when switching immunosuppression from CNIs to belatacept late after transplantation, an immediate increase in eGFR has been shown consistently [8,9]. This suggests that direct vasoconstriction is reversed in patients even in the presence of chronic CNI toxicity.

B-Mode ultrasound (US) and color-coded duplex ultrasound (CCDS) including measurement of resistance indices (RI) remain the primary imaging modalities in follow-up (FU) of renal grafts. In general, CCDS depicts the macrovascularization with limitations on assessment of cortical vascularity due to their small vessels and low arterial flow. Ultrasound contrast agents (UCA) and dynamic depiction of the renal microvascularity overcome the physical limitation of CCDS by visualization of both the macro- and the microvasculature. After intravenous injection, the main branches enhance first, rapidly followed by the segmental to arcuate and interlobular arteries, and finally complete cortical enhancement within seconds. Due to its dynamic contrast imaging, contrast-enhanced ultrasound (CEUS) is additionally used for identification of early vascular complications and whole-organ perfusion (such as parenchymal infarction), or inflammatory complications (such as abscess) with a higher sensitivity by depiction of microvascularization [10,11].

Due to its superficial localization and small movement during breathing, the renal graft shows optimal requirements for dynamic perfusion imaging in standardized longitudinal planes using a post-processing tool. In order to study the suitability of CEUS to assess the effects of CNI cessation on kidney allograft perfusion, we performed standardized CEUS in patients who underwent the switch from CNIs to belatacept for clinical indication. Additionally, we compared the suitability of convex and linear probes when performing standardized CEUS in renal allografts.

## 2. Methods

### 2.1. Patient Selection, Switch of Immunosuppression, and Clinical Data

We enrolled eleven patients who underwent the switch of immunosuppressive regimen from CNIs to belatacept for clinical indication. Belatacept was administered 5 mg/kg every 14 days for 8 weeks, and then every 4 weeks thereafter. CNI treatment was tapered as follows: 100% on day 1, 40–60% on day 15, 20–30% on day 23, and none on day 29 and beyond.

Donor characteristics evaluated included age, sex, and living versus deceased donation. Recipient characteristics evaluated included age, sex, cause of chronic kidney failure, type of dialysis, duration of dialysis, induction immunosuppressive regimen, early graft function, time since transplantation, cause of switch of immunosuppression, arteriolar hyalinosis (ah) grade according to Banff 2017 classification in the latest kidney allograft biopsy, and baseline immunosuppression. Delayed graft function was defined as the need of dialysis within seven days after transplantation. Recipient serum creatinine, microalbuminuria, and kidney biopsy results were examined at baseline (day of first measurement) and m6 (day of last measurement).

### 2.2. Imaging Protocol

B-mode ultrasound of all renal transplants was performed using a convex array transducer to assess organ position and standardized longitudinal planes. All convex transducers employed in study patients were required to be for abdominal use with a frequency range of 1–6 MHz. CEUS examinations were performed using high-end ultrasound systems (Aplio i900, Canon Medical Systems Corporation, Tochigi, Japan; Acuson Sequoia, Siemens Healthineers, Mountain View, CA, USA; GE Logiq E10, GE Healthcare, Chicago, IL, USA) with state-of-the-art CEUS-specific protocols. A low mechanical index (<0.1) was used to avoid early microbubble destruction, especially in the near field. A bolus of 1.6 mL of a second-generation ultrasound contrast agent (SonoVue^®^, Bracco Imaging, Milan, Italy) was injected and a cine loop of 60 s was stored to assess inflow and washout. After a time delay of 10 min, the examination was repeated with a linear array transducer using a penetration depth of up to 5 cm focused on the superficial renal cortex. A cine loop of 60 s after a second injection of 1.6 mL SonoVue^®^ was repeated after full washout of the ultrasound contrast agent of the first scan. 

### 2.3. Perfusion Quantification

**Figure 1:**
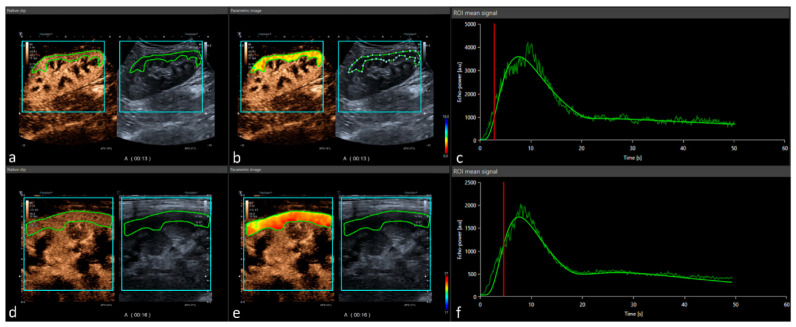
Post-processing contrast-enhanced ultrasound perfusion analysis: CEUS DICOM loops are implemented in the software tool Vuebox^®^ (Bracco Imaging) and analyzed using a demarcation ROI (turquoise) and analysis ROI (green). Represented data show a case of convex probe (**a**) with corresponding color-coded map, (**b**) peak enhancement, and (**c**) time–intensity curve analysis. After full elimination, the procedure was repeated with a linear probe (**d**) showing the equal color-coded map, (**e**) peak enhancement, and (**f**) time–intensity curve analysis.

Quantification of perfusion imaging on CEUS was performed using the post-processing software tool VueBox^®^ (Bracco Suisse SA-Software Applications, Geneva, Switzerland) by using uncompressed DICOM cine loops. After initial software calibration (ultrasound transducers, image presets, and gain adaption), regions of interest (ROI) were manually placed in the renal cortex by an experienced radiologist specialized in CEUS. The ROI was placed in the renal cortex excluding the medullar pyramids (Figure 1). ROI position did not change during the entire clip. If needed, motion compensation was used to compensate minimal movement during the 60 s cine loop. Quantitative perfusion assessment included time-related parameters such as rise time (RT), time to peak (TTP), mean transit time (mTT), and fall time (FT). Intensity parameters were peak-enhancement (PE), wash-in area under the curve (WiAUC), wash-out area under the curve (WoAUC), wash-in rate (WiR), wash-out rate (WiR), wash-in–wash-out area under the curve (WiWoAUC), and wash-in perfusion index (WiPI). All parameters were evaluated on cine loops using both transducer techniques (both convex and linear transducer). Time intensity parameters were started by depiction of the first microbubble in the delamination area to avoid potential differences of inflow time due to potentially impaired cardiac output. Intensity parameters are linearized signals of brightness of the pixels fitted to a predetermined time interval resulting in standardized time–intensity curves.

### 2.4. Statistical Analysis

A dependent t-test was applied to compare after testing for normality using Shapiro–Wilk’s method. A two-sided significance level of α 0.05 was deemed appropriate to indicate statistical significance. All statistical analyses were performed using the SPSS software (IBM Corp. Released 2016. IBM SPSS Statistics for Windows, Version 27.0. Armonk, NY, USA: IBM Corp) and “R” version 4.1.1. 

## 3. Results

### 3.1. Patient Characteristics

Eleven patients were enrolled with the mean age at inclusion being 47 ± 13 years. The immunosuppressive regimen was switched 7.8 years after transplantation on average, with 8/11 patients receiving tacrolimus and 3/11 patients receiving cyclosporine before CNI cessation. All patients except for one, who was on dual antiplatelet therapy, underwent kidney allograft biopsy before switching to belatacept. Severe arteriolar hyalinosis (ah3) as a sign of chronic CNI toxicity was found in 8/11 patients. Additionally, one patient showed moderate arteriolar hyalinosis (ah2), one patient showed acute tubular CNI toxicity (isometric tubular cell vacuolization) but no chronic vessel changes (ah0), and one patient—for whom no biopsy could be obtained—received rescue switch due to marginal kidney function. Additional patient characteristics are shown in Table 1.

### 3.2. Renal Function, Albuminuria, and Acute Rejections

**Figure 2:**
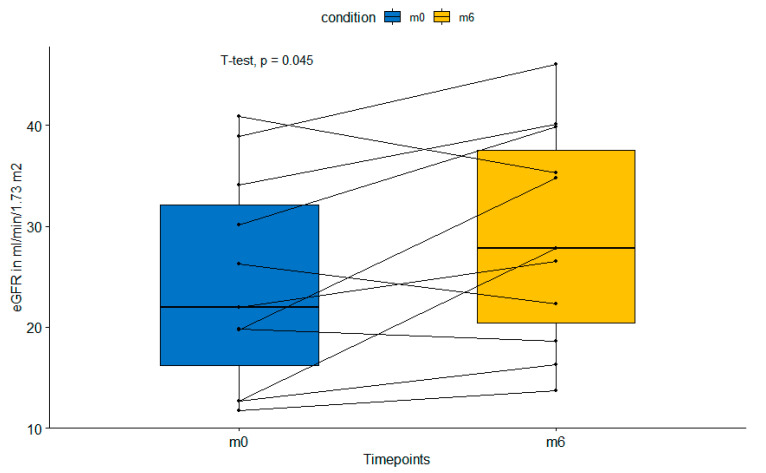
Paired boxplot showing significant increase in estimated glomerular filtration rate from baseline (m0) to 6 months (m6) after switch to belatacept. eGFR—estimated glomerular filtration rte.

All patients had a functioning graft at the end of the study period. eGFR increased significantly from 24.4 ± 10 mL/min/1.73 m^2^ at baseline to 29.2 ± 10.3 mL/min/1.73 m^2^ at the end of the study period (*p* = 0.045) as shown in Figure 2. Simultaneously, the average albumin–creatinine ratio changed from 0.57 ± 1.0 to 0.80 ± 1.3 after the switch to belatacept (*p* = 0.14), which is due to increased glomerular perfusion pressure after the reversal of afferent vasoconstriction. No acute rejection episodes were observed during the observation period.

### 3.3. CEUS Results

The quality of fit on the perfusion imaging was high in the convex (baseline 95.6% and FU 96.6%) and linear transducer (baseline 97.7% and FU 96.6%). Mean ROI size in perfusion assessment was 9.4 cm^2^ (baseline) vs. 9.2 cm^2^ (FU) for CEUS with a convex transducer and 3.5 vs. 2.6 cm^2^ for CEUS using a linear transducer. As represented in Table 2, neither the intensity parameters nor the time-related parameters depict a significant difference between baseline and FU examination. Although not reaching significant differences, intensity parameters revealed a slight increase on FU in both convex and linear transducers, while time-related parameters showed only minor changes with a decrease on the convex transducer and an increase on the linear transducers. When analyzing patients, whose eGFR improved (8/11) and those with declining eGFR (3/11) separately, no significant changes were detected either (data not shown).

## 4. Discussion

In this pilot study including eleven kidney transplant patients, standardized CEUS perfusions analysis did not show a statistically significant change of renal perfusion after switching immunosuppression from CNIs to belatacept for clinical indication. This is the first study that systematically investigates the suitability of CEUS to assess microvascular perfusion changes in KTR in vivo. After CNI cessation, an increased microvascular perfusion is expected due to the reversal of CNI-induced vasoconstriction of the afferent arterioles. This increase in perfusion is displayed by the average eGFR increase of 4.8 mL/min/1.73 m^2^ (16%) after CNI cessation, which is comparable to other studies [8,9].

### 4.1. Perfusion Imaging on CEUS

Over the last decade, the clinical use of CEUS has consistently increased by implementation in guidelines. While liver imaging represents the major clinical application, which is denoted by a separate guideline of hepatic imaging, all extrahepatic indications are implemented within one guideline [11,12]. In renal imaging, CEUS is primary used for assessment of cystic renal lesions or organ perfusion to depict microvascular changes such as ischemia (infarction, acute renal cortical necrosis) or major vessel problems [11].

Using newer software tools or post-processing applications, perfusion imaging shows the possibility of parametric imaging with the assessment of renal perfusion in real time. Thus, quantitative time-related or intensity-related parameters are presented by time–intensity curve analysis. CEUS is an established tool in the assessment of focal lesions, but newer studies have focused on the evaluation of whole-organ perfusion. In a recently published study, El-Bandar and colleagues demonstrated only a minor correlation of CEUS parameter and split renal function assessed on preoperative scintigraph evaluation in living kidney donors [13]. The study results presented an association of signal intensity parameters on CEUS with kidney function only in normal-weight individuals. The authors concluded that body mass index (BMI) may be a potential confounder of renal perfusion quantification. 

Quantification of whole-organ perfusion was also analyzed by Chen and colleagues to assess the renal function in children with ureteropelvic junction obstruction. The study group investigated CEUS perfusion imaging in 102 kidneys of 51 children with unilateral disease. Similar to our study findings, no significant difference in perfusion parameter and their AUC was detected between the diseased and healthy kidneys [14]. Harrois et al. denoted short-term changes in renal blood flow assessed by CEUS [15]. Patients with septic shock on intensive care unit showed decreased renal cortical perfusion represented by lower perfusion index (PI) and higher mean transit time compared to patients without. They noted changes within three days depicted by improvement of PI compared to baseline examination. Notably, they also observed a wide range of PI values in the cohort of patients with septic shock, which is comparable to our findings by the use of convex transducers. Schneider et al. investigated potential changes of renal perfusion on CEUS after the increase in mean arterial pressure due to noradrenaline infusion in critically ill patients. The study group denoted no changes on perfusion parameter after increase in mean arterial pressure from baseline (60–65 mmHg) to follow-up (80–85 mmHg) [16]. Similar to our results, only a minority of perfusion (AUC) parameters showed a minor correlation of glomerular filtration rate in patients with diabetic kidney damage (r = 0.472, *p* = 0.01), while time-related parameters such as time-to-peak did not (r = 0.262, *p* = 0.177) [17].

In pre-clinical studies, both RI measurements and CEUS perfusion parameters showed only partially promising results in perfusion changes during hypoxia [18]. In a group of 12 piglets, hypoxia was associated with decreased arteriovenous transit time with significant changes in cortical perfusion compared to a control group. Cortical and medullar perfusion were affected differently, showing an increase in medullary time-related perfusion parameters—indicating a reduction in medullary blood flow velocity—and only a significant decrease in cortical perfusion index, probably because of a reduction in cortical blood volume. Overall, cortical perfusion showed no statistically significant changes in time-related parameters observed during hypoxia. On the contrary, Li et al. have demonstrated a significant increase in time-related parameters (arrival time, time-to-peak, AUC) in rabbits with ischemia–reperfusion injury as a result of impaired renal cortex blood perfusion [19]. 

### 4.2. Technical Aspects

Data on the evaluation of perfusion imaging using a high-frequency linear transducer and low-frequency convex transducer are rare. Agreement among three readers regarding the evaluation of the degree of enhancement, internal homogeneity, and the perfusion defects in breast lesions varied between fair and substantially better for the evaluation with linear transducers [20]. The intra-operative use of high-frequency CEUS may depict additional findings on perfusion pattern and therefore improve the visualization of tumour lesions in the liver [21]. In our study, perfusion parameters on CEUS using convex transducers showed a wider range of metric perfusion parameters compared to linear transducers. This might be a technical bias due to the lower resolution and impaired frames per second. Although linear transducers show a smaller area of interest, the cortical perfusion may be more accurate due to a higher frames per second and consecutively improved depiction of blood flow. In contrast, Wang et al. showed improved diagnostic performance in breast lesions using convex probes by the employment of CEUS in detection of malignant tumours [22]. Overall, representative study data on a direct comparison in whole-organ perfusion—especially renal transplant—is currently missing.

Due to an immense wide range of perfusion parameters (especially intensity parameters) using the convex probe, the linear probe showed better suitability with smaller range and more robust data. Although a smaller field of view, wash-in/-out characteristics may demonstrate improved visualization on the cortical level. In conclusion, for dynamic assessment of cortical perfusion, the linear probe may be more adequate and should be used in detailed follow-up studies. While further investigations with larger sample sizes may detect significant changes for certain CEUS parameters in a comparable experimental setup, this pilot study indicates that routine CEUS is not able to detect slight changes in microvascular perfusion with sufficient accuracy to be transferred to clinical practice in the near future. Since most patients received late conversion to belatacept in the present study, arteriolar hyalinosis may be more pronounced and less reversible in comparison to patients who receive early conversion to belatacept in the first post-transplant year. It would be interesting to study CEUS in patients with early conversion to belatacept, where improvement in microvasculature is suspected to be more pronounced.

## 5. Limitations

The major technical limitation of perfusion analysis was the missing standardization. Although all ROIs are placed equally, the parametric intensity values are not comparable between patients. Likewise, the wide spread of values regarding intensity parameters is difficult to compare in one patient. This may be due to different US parameters, although gain was not changed and calibrated on perfusion analyses. Thus, in the discussion we focused on the more robust parametric values assessed by the linear transducer. The smaller scan field and superficial approach were associated with a smaller range of values. Moreover, the use of different US machines may influence all values, although post-processing analysis should allow comparison of different US machines and probes.

A methodological limitation which could be improved in following studies is the missing assessment of renal microvascular changes by follow-up biopsy. Such follow-up biopsies could add further evidence that CNI cessation improved microvascular perfusion besides eGFR improvement. On the other hand, histopathological changes in kidney allograft biopsies are patchy, and the effect of CNI withdrawal on microvascular diameter can be expected to be mostly functional and small. Therefore, changes of microvasculature can be undetectable by routine biopsy, and functional parameters such as eGFR may be better suited. Lastly, CEUS is not well established in kidney allograft, which limits its applicability to detect small changes in microvascular perfusion.

## Figures and Tables

**Table 1:** Demographics and baseline characteristics of 11 kidney transplant recipients, who underwent switch to belatacept from CNI-based immunosuppressive regimen due to clinical indication. No.—number; Rec.— recipient; KFRT—kidney failure requiring renal replacement therapy; IgAN—IgA nephropathy; HUS—hemolytic uremic syndrome; ADPKD—autosomal dominant polycystic kidney disease; HD—hemodialysis; PD—peritoneal dialysis; dec.—deceased; comp.—compatibility; incomp.—incompatible; CIT—cold ischemia time; IS—immunosuppression; CNI—calcineurin inhibitor; KTx—kidney transplantation; ATN—acute tubular necrosis; ah—arteriolar hyalinosis according to Banff 2017 classification; caABMR—chronic active antibody mediated rejection; CAD—coronary artery disease; DM—diabetes mellitus; py—pack years; KDPI—kidney donor profile index; KDRI—kidney donor risk index.

Patient No.	Rec. Age (Years) /Sex	Cause of KFRT	Dialysis Modality/Year on Dialysis	Donor Age Years/Sex	Living vs. dec. Donor/AB0 Comp.	CIT (min)	Induction Therapy	Baseline IS (Daily CNI dose)	Time after KTx (years)	Cause for IS Switch/Ah grade	CAD/DM/Smoking Status	KDPI/KDRI	Donor Cause of Death/Comorbid Conditions
1	34/female	IgAN	HD/11	49/female	Dec.	342	Basiliximab	Tacrolimus, extended-release 10 mg + MPA + Steroid	1	ATN due to chronic hypotension/ah3	no/no/active (30 py)	38%/0.89	Subarachnoid hemorrhage/arterial hypertension
2	34/male	IgAN	PD/2	33/female	Living/AB0 comp.	165	Basiliximab	Tacrolimus, immediate-release 8 mg + MPA + Steroid	5	ah3	no/no/active (5 py)	22%/0.75	None/Hypercholesterinemia
3	32/female	HUS	n/a	43/female	Living/AB0 comp.	140	Basiliximab	Tacrolimus, immediate-release 6 mg + MPA + Steroid	11	ah3	no/no/never	33%/0.85	None/none
4	57/male	DM	HD/8	64/male	Dec.	543	Basiliximab	Tacrolimus, extended-release 7 mg + MPA + Steroid	1	no biopsy—suspected CNI toxicity	yes/yes/never	94%/1.73	Intracerebral hemorrhage/arterial hypertension, severe arteriosclerosis
5	32/male	IgAN	HD/1	47/male	Living/AB0 comp.	185	Basiliximab	Tacrolimus, immediate-release 8 mg + MPA + Steroid	8	TMA from CNI or caAMR/ah3	no/no/never	11%/0.66	None/none
6	69/male	ADPKD	HD/1	61/female	Living/AB0 incomp.	170	Rituximab, Basiliximab	Tacrolimus, immediate-release 3 mg + MPA + Steroid	7	ATN from CNI toxicity/ah3	no/no/never	71%/1.23	None/arterial hypertension
7	53/male	Alport	PD/1	45/male	Dec.	674	Basiliximab	Cyclosporin 200 mg + MPA	13	ah2	No/yes/former (20 py)	30%/0.82	Head trauma/none
8	39/male	IgAN	n/a	54/female	Living/AB0 comp.	170	Basiliximab	Cyclosporin 170 mg + MPA + low-dose Steroid	10	ah3	no/no/never	63%/1.12	None/Cholecystectomy
9	53/male	IgAN	PD/1	56/female	Living/AB0 comp.	123	Basiliximab	Cyclosporin 250 mg + MPA + low-dose Steroid	16	ah3	no/no/never	44%/0.94	None/urinary tract infections
10	53/female	unknown	HD/11	55/female	Dec.	520	Basiliximab	Tacrolimus, slow-release 7 mg + MPA + Steroid	4	ATN from acute CNI toxicity/ah0	No/no/never	93%/1.65	Subarachnoid hemorrhage/HCV infection
11	62/female	ADPKD	HD/3	52/male	Living/AB0 incomp.	168	Basiliximab	Tacrolimus, immediate-release 2 mg + MPA + low-dose Steroid	10	ah3	No/yes/former (50 py)	25%/0.77	APC resistance

**Table 2:** Median difference is represented as the percentual change between baseline US (timepoint m0) and FU (timepoint m6) using convex transducer and linear transducers collected by the same US protocol. Corresponding *p*-value is calculated using paired *t*-test.

Contrast-Enhanced Ultrasound				
	Convex	*p*-Value	Linear	*p*-Value
**meanLin**	+1523.4%	0.187	+50.5%	0.391
**PE**	+1888.2%	0.212	+48.2%	0.614
**WiAUC**	+1743.5%	0.200	+49.5%	0.531
**RT (s)**	−6.1%	0.297	+13.0%	0.727
**mTT (s)**	+48.8%	0.301	+35.0%	0.638
**TTP (s)**	−3.0%	0.317	+8.1%	0.927
**WiR**	+2056.3%	0.201	+63.8%	0.501
**WiPI**	+1876.9%	0.209	+48.3%	0.572
**WoAUC**	+1661.2%	0.173	+44.5%	0.345
**WiWoAUC**	+1680.8%	0.182	+44.4%	0.391
**FT (s)**	−6.3%	0.428	+22.0%	0.655
**WoR**	+2324.6%	0.224	+72.9%	0.913

Abbreviations: meanLin denotes mean linearized intensity signal; PE, peak enhancement; WiAUC, wash-in area under the curve; RT, rise time; mTT, mean transit time; TTP, time to peak; WiR, wash-in rate; WiPi, wash-in perfusion index; WoAUC, wash-out area under the curve; WiWoAUC, wash-in/wash-out area under the curve; FT, fall time; WoR, wash-out rate.

## Data Availability

Data can be made available on reasonable request to the corresponding author.
